# Intelligence and Brain Efficiency: Investigating the Association between Working Memory Performance, Glutamate, and GABA

**DOI:** 10.3389/fpsyt.2017.00154

**Published:** 2017-09-15

**Authors:** Anouk Marsman, René C. W. Mandl, Dennis W. J. Klomp, Wiepke Cahn, René S. Kahn, Peter R. Luijten, Hilleke E. Hulshoff Pol

**Affiliations:** ^1^Brain Center Rudolf Magnus, Department of Psychiatry, University Medical Center Utrecht, Utrecht, Netherlands; ^2^Department of Radiology, University Medical Center Utrecht, Utrecht, Netherlands

**Keywords:** ^1^H-MRS, 7 T, gamma-aminobutyric acid, glutamate, intelligence

## Abstract

Intelligence is a measure of general cognitive functioning capturing a wide variety of different cognitive functions. It has been hypothesized that the brain works to minimize the resources allocated toward higher cognitive functioning. Thus, for the intelligent brain, it may be that not simply more is better, but rather, more efficient is better. Energy metabolism supports both inhibitory and excitatory neurotransmission processes. Indeed, in glutamatergic and GABAergic neurons, the primary energetic costs are associated with neurotransmission. We tested the hypothesis that minimizing resources through the excitation–inhibition balance encompassing gamma-aminobutyric acid (GABA) and glutamate may be beneficial to general cognitive functioning using 7 T ^1^H-MRS in 23 healthy individuals (male/female = 16/7, 27.7 ± 5.3 years). We find that a higher working memory index is significantly correlated with a lower GABA to glutamate ratio in the frontal cortex and with a lower glutamate level in the occipital cortex. Thus, it seems that working memory performance is associated with the excitation–inhibition balance in the brain.

## Introduction

Intelligence is a measure of general cognitive functioning capturing a wide variety of different cognitive functions (1). Intelligence has long been (albeit modestly) associated with brain size (2–4). More recently, intellectual functioning has been implicated in brain functioning (5) and in the efficiency of the functional (6) and structural brain network (7). Regional structural differences in relationship to intelligence have been demonstrated in several studies in healthy individuals (2, 3, 8) and in individuals with local brain lesions (9). However, it is not known how the human brain handles complex cognitive tasks while being such an expensive organ to operate, utilizing some 20% of all oxygen taken in and 25% of all glucose produced while representing only about 2% of the body’s weight (10, 11). Indeed, it has been hypothesized that the brain works to minimize the resources allocated toward higher cognitive functioning (12). Thus, for the intelligent brain, it may be that not simply more is better, but rather more *efficient* is better.

Glutamate (Glu) and gamma-aminobutyric acid (GABA) are the major excitatory and inhibitory neurotransmitters in the central nervous system. In both glutamatergic and GABAergic neurons, the primary energetic costs are associated with neurotransmission, and the energetic needs of these neurons dominate the cerebral cortex energy requirements (13, 14). In the resting awake state, 80% of energy used by the brain supports events associated with neuronal firing and cycling of GABA and glutamate, and in the actively awake individual, the change in energy (and its coupled activity) induced by stimulation during task performance is very small in comparison to its baseline value (15). Thus, minimizing resources through the inhibition–excitation balance encompassing GABA and glutamate may be beneficial to general cognitive functioning. While general intellectual functioning has been related to brain neurochemistry in several studies, measuring largely positive associations with the brain metabolite *N*-acetyl aspartate (NAA) (16, 17), a marker of neuronal integrity (18, 19), these measures do not provide information on the inhibition–excitation balance. To obtain such information, one needs to reliably measure both glutamate and GABA levels, which is not an easy task using MR scanners operating at conventional magnetic field strengths.

Proton magnetic resonance spectroscopy (^1^H-MRS) at a field strength of 7 T has an increased sensitivity and spectral resolution. For instance, at 7 T, it is now possible to adequately separate the glutamate and glutamine signals resulting in a higher accuracy of glutamate measurement (20). Despite the increased sensitivity, measurement of GABA is not straightforward because of its low concentration compared to other brain metabolites and its obscured signal due to overlapping signals of higher intensity. To overcome this problem, spectral editing techniques can be applied to isolate the GABA signal (21). Using these methods, we were able to show that higher cognitive functioning was associated with lower GABA levels in the prefrontal cortex in patients with schizophrenia (22).

We explored possible associations between the excitation–inhibition balance in the brain and intelligence in the prefrontal cortex and in the occipital cortex. For this purpose, we measured GABA and glutamate levels using ^1^H-MRS at a magnetic field strength of 7 T in healthy adults. While a whole brain approach would be ideal for studying intelligence, it is currently not feasible to measure GABA and glutamate reliably at a whole brain level using MRS. Therefore, we chose the prefrontal and occipital cortex because the prefrontal cortex has long been found to be important to general cognitive functioning and because the occipital cortex could be considered as a control area. This selection of areas has limitations. Indeed, the frontal lobes are often considered the primary focus of human intelligence (23, 24). However, the parieto-frontal integration theory for intelligence (25–27), an association with the whole brain network with intelligence (6), and association of intelligence level with cortical thickness change in many brain areas, including prefrontal and occipital cortices (28) suggest that many different parts of the brain may be involved in intelligence. Moreover, the occipital cortex was also involved in earlier studies [e.g., Ref. (23)]. We hypothesized that the prefrontal cortex is essential for intelligence and thus expect an association with GABA and glutamate and intelligence level in this brain area and not in the occipital cortex in this exploratory study.

## Materials and Methods

### Subjects

A total of 23 healthy individuals (16 males/7 females) participated in the study. Participants had no major psychiatric or neurological history, no history of drug or alcohol abuse, and no first-degree relatives with psychiatric or neurological disorders. The study was approved by the Medical Ethics Committee of the University Medical Center Utrecht, The Netherlands, and performed according to the directives of the Declaration of Helsinki (amendment of Seoul, 2008). Participants provided written informed consent prior to the examination. Mean (SD) age was 27.7 (5.3) years, and average completed years of education was 14.1 ± 2.1 years.

### Cognitive Assessment

All participants underwent a general cognitive assessment using the full Wechsler Adult Intelligence Scale (WAIS–III-NL) (29). The total intelligence quotient (TIQ) as well as the verbal (VIQ) and performance (PIQ) intelligence quotients, the perceptual reasoning index (PRI), verbal comprehension index (VCI), and the working memory index (WMI) were measured.

### MR Acquisition

All investigations were performed on a 7 T whole body MR scanner (Philips, Cleveland, OH, USA). A birdcage transmit head coil was used in dual transmit driven by 2 × 4 kW amplifiers, in combination with a 32-channel receive coil (both Nova Medical, Inc., Burlington, MA, USA).

For anatomical reference and gray and white matter tissue classification, a T_1_-weighted magnetization prepared rapid gradient echo sequence was obtained (450 slices, slice thickness = 0.8 mm, TR = 7 ms, TE = 3 ms, flip angle = 8°, FOV = 250 mm × 200 mm × 180 mm, 312 × 312 acquisition matrix, SENSE factor 2.7, scan duration = 408 s).

**Figure 1:**
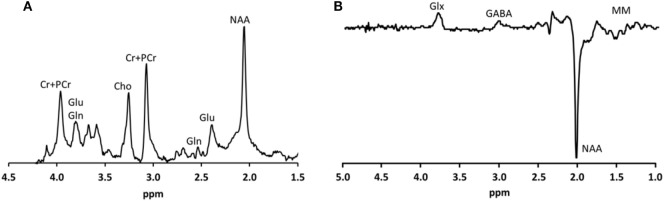
Typical metabolite spectra **(A)** as recorded using the sLASER sequence and **(B)** as recorded using the MEGA-sLASER sequence.

**Figure 2:**
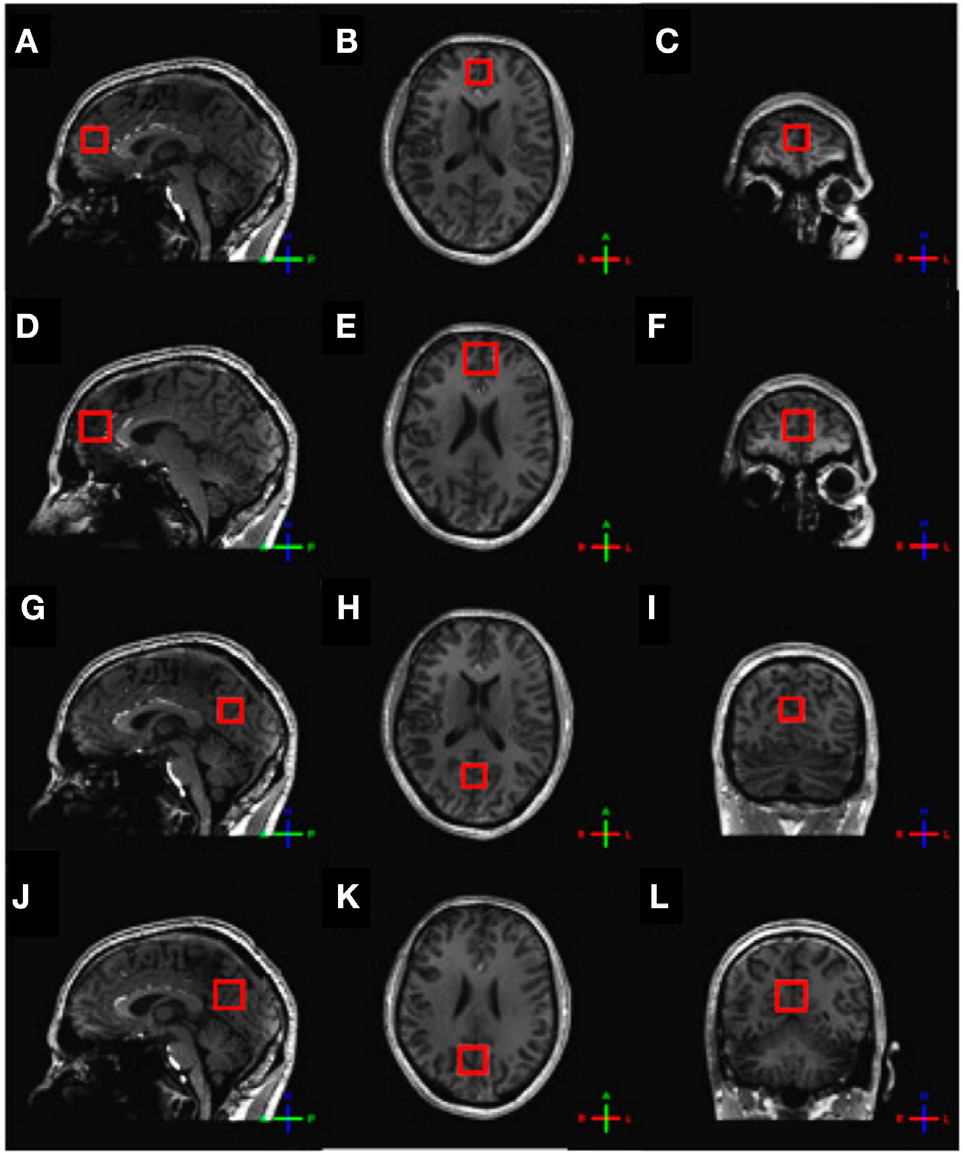
Voxel placement. **(A)** Frontal sLASER voxel, sagittal view; **(B)** frontal sLASER voxel, axial view; **(C)** frontal sLASER voxel, coronal view; **(D)** frontal MEGA-sLASER voxel, sagittal view; **(E)** frontal MEGA-sLASER voxel, axial view; **(F)** frontal MEGA-sLASER voxel, coronal view; **(G)** occipital sLASER voxel, sagittal view; **(H)** occipital sLASER voxel, axial view; **(I)** occipital sLASER voxel, coronal view; **(J)** occipital MEGA-sLASER voxel, sagittal view; **(K)** occipital MEGA-sLASER voxel, axial view; **(L)** occipital MEGA-sLASER voxel, coronal view [from Ref. (22)].

For the assessment of glutamate, ^1^H-MRS experiments were conducted using a sLASER sequence (semi-localized by adiabatic selective refocusing; TE = 28 ms, 32 averages, TR = 5 s) (Figure 1A). Voxels (2 cm × 2 cm × 2 cm) were located in the medial prefrontal and medial occipital lobe (Figure 2). Non-water-suppressed spectra were obtained for quantification (carrier frequency was set to the chemical shift of H_2_O, acquisition time = 10 s).

GABA-edited ^1^H-MRS experiments were conducted using a MEGA-sLASER sequence (TE = 74 ms, 64 averages, TR = 4 s) (Figure 1B) (31). Voxels (2.5 cm × 2.5 cm × 2.5 cm) were located in the medial frontal and medial occipital region (Figure 2). Prior to the MRS exams, second order B_0_ shimming was applied using the FASTERMAP algorithm at the voxel of interest (30, 32). Second, at this location, a high B_1_ field was generated to minimize chemical shift displacement artifacts (33). The highest possible B_1_ field was generated by optimizing the phase of both transmit channels to locally assure constructive B_1_ interferences (34).

### Spectral Fitting and Quantification

Retrospective phase and frequency alignment was performed on all data sets of each measurement (35). Fitting of the sLASER spectra was performed with LCModel-based software implemented in Matlab (36), which uses *a priori* knowledge of the spectral components to fit metabolite resonances (37). The following 16 metabolites and a measured macromolecular baseline (38) were fitted to the spectra: acetate, aspartate, choline (Cho), phosphorylcholine, glycerophosphorylcholine, phosphorylethanolamine, creatine (Cr), phosphocreatine (PCr), NAA, *N*-acetyl aspartyl glutamate, GABA, Glu, glutamine (Gln), glutathione (GSH), myo-inositol (mIns), and taurine (Tau). Glutamate levels were estimated using the water signal as an internal reference and calculated as follows:

$$\begin{array}{l} {}[\text{met}]=\;\left(\frac{\frac{{\text{signal}}_{\text{met}}}{{\text{signal}}_{\text{water}}}⋆({\text{volGM}}^{⋆}{\text{[water}}_{\text{GM}}\text{]+ }{\text{volWM}}^{⋆}{\text{[water}}_{\text{WM}}{\text{]+volCSF}}^{⋆}{\text{[water}}_{\text{pure}}\text{]})}{\text{volGM+volWM}}\right) \end{array}$$

where [met] is the metabolite concentration, signal_met_ is the fitted signal intensity of the metabolite, accounting for the number of protons, and signal_water_ is the fitted signal intensity of water, accounting for the number of protons; volGM, volWM, and volCSF are, respectively, the gray matter fraction, white matter fraction, and cerebrospinal fluid (CSF) fraction in the voxel; and [water_GM_], [water_WM_], and [water_pure_] are, respectively, the water concentration in gray matter, white matter, or CSF. For determining the contribution of gray matter, white matter, and CSF of each voxel, the software package SPM8 was used to segment the T_1_-weighted image. In the T_1_-weighted image, the position of the ^1^H-MRS voxel was determined, after which the amount of gray matter, white matter, and CSF in the ^1^H-MRS voxel was computed. To account for differences in transverse relaxation between water and metabolites, a correction was applied based on reported T_2_ values at 7 T of 47 ms on average for water and 107 ms assumed for the metabolites (39). Statistical analysis of the gray and white matter fractions in the frontal and occipital MEGA-sLASER (GABA/Cr) and sLASER (glutamate) revealed correlations >0.95 for both gray and white matter fractions in the two voxels.

Fitting of the MEGA-sLASER spectra was performed by frequency-domain fitting of the GABA and creatine resonances to a Lorentzian line-shape function in Matlab. GABA levels were expressed as the ratios of their peak areas relative to the peak areas of the creatine resonance (GABA/Cr).

### Statistical Analysis

Statistical analyses were performed using SPSS 21.0 (2012, Chicago, IL, USA). Data were controlled for their normality of the distributions. No transformations for correction were needed. To evaluate differences in metabolite concentrations and gray and white matter fractions between the frontal and occipital areas, paired *t*-tests were done. To evaluate associations between brain metabolite levels with general intelligence measures, partial correlation coefficients were done with corrections for age, sex, and for gray and white matter fractions (40). The partial correlation between IQ and metabolite levels was defined as the correlation between the residuals of IQ and metabolite levels resulting from the linear regressions of IQ and metabolite levels with the controlling variables, i.e., age, sex, and gray and white matter fractions of the sLASER and/or MEGA-sLASER voxels. To correct for multiple comparisons, while taking the considerable dependency among intelligence quotient measures and the exploratory nature of the study into account, we restricted the significance level of the correlations to *p* ≤ 0.01.

## Results

### Intelligence Measures

The mean (SD) of the TIQ was 108 (13), the verbal intelligence quotient (VIQ) was 109 (12), the performance intelligence quotient (PIQ) was 107 (14), the VCI was 111 (13), the PRI was 108 (15), and the WMI was 105 (12) (Table 1).

**Table 1:** Intelligence and brain metabolites in healthy individuals.^a^

Intelligence	(Sub) test score	Mean (SD)	Min	Max
	Total IQ	108 (13)	82	131
	Verbal IQ	109 (12)	82	128
	Performance IQ	107 (14)	78	127
	Verbal comprehension index	111 (13)	91	132
	Perceptual reasoning index	108 (15)	79	129
	Working memory index	105 (12)	86	124

**^1^H-MRS sequence**	**Metabolite**	**Prefrontal [mean (SD)]**	**Occipital [mean (SD)]**	**Paired ***t***-test**

MEGA-sLASER	GABA/Cr ratio	0.14 (0.03)	0.13 (0.03)	ns
	Gray matter (%)	68.1 (11.3)	68.0 (11.9)	ns
	White matter (%)	24.0 (11.8)	27.7 (13.2)	ns
sLASER	Glutamate (mM)	8.65 (1.14)	8.48 (1.26)	ns
	Gray matter (%)	71.4 (14.3)	70.1 (12.7)	ns
	White matter (%)	19.5 (15.6)	25.2 (14.6)	*p* < 0.05^b^

*^a^Uncorrected data based on two separate ^1^H-MRS measurements and T_1_-weighted volume measurements performed in two brain areas (medial prefrontal and medial occipital). For assessment of GABA/Cr ratios, a MEGA-sLASER sequence was performed and successfully completed in 19 individuals in the medial prefrontal cortex and in 18 individuals in the medial occipital cortex. A sLASER sequence was performed for assessment of glutamate and successfully completed in 18 individuals in the medial prefrontal cortex and in 17 individuals in the medial occipital cortex. Volume data are based on 21 individuals except for the occipital MEGA-sLASER voxel volumes, which are based on 18 individuals*.

*^b^Following corrections for age and sex, this difference was no longer significant*.

### Metabolite Concentrations in the Frontal and Occipital Areas

Because of poor spectral quality as established by a Cramér-Rao lower bound of more than 20% and visual inspection, some data were excluded from the study. Frontal MRS results are based on 18 subjects and occipital MRS results are based on 17 subjects. Frontal GABA-edited MRS results are based on 19 subjects and occipital GABA-edited MRS results are also based on 19 subjects.

Paired *t*-tests for differences in metabolite concentrations and gray and white matter fractions between the frontal and occipital areas revealed no significant differences except for a higher white matter fraction in the occipital sLASER voxel, but this finding did not survive the analysis after correction for age and sex (Table 1).

### Brain Metabolites, Gray and White Matter Fractions, and Intelligence

**Figure 3:**
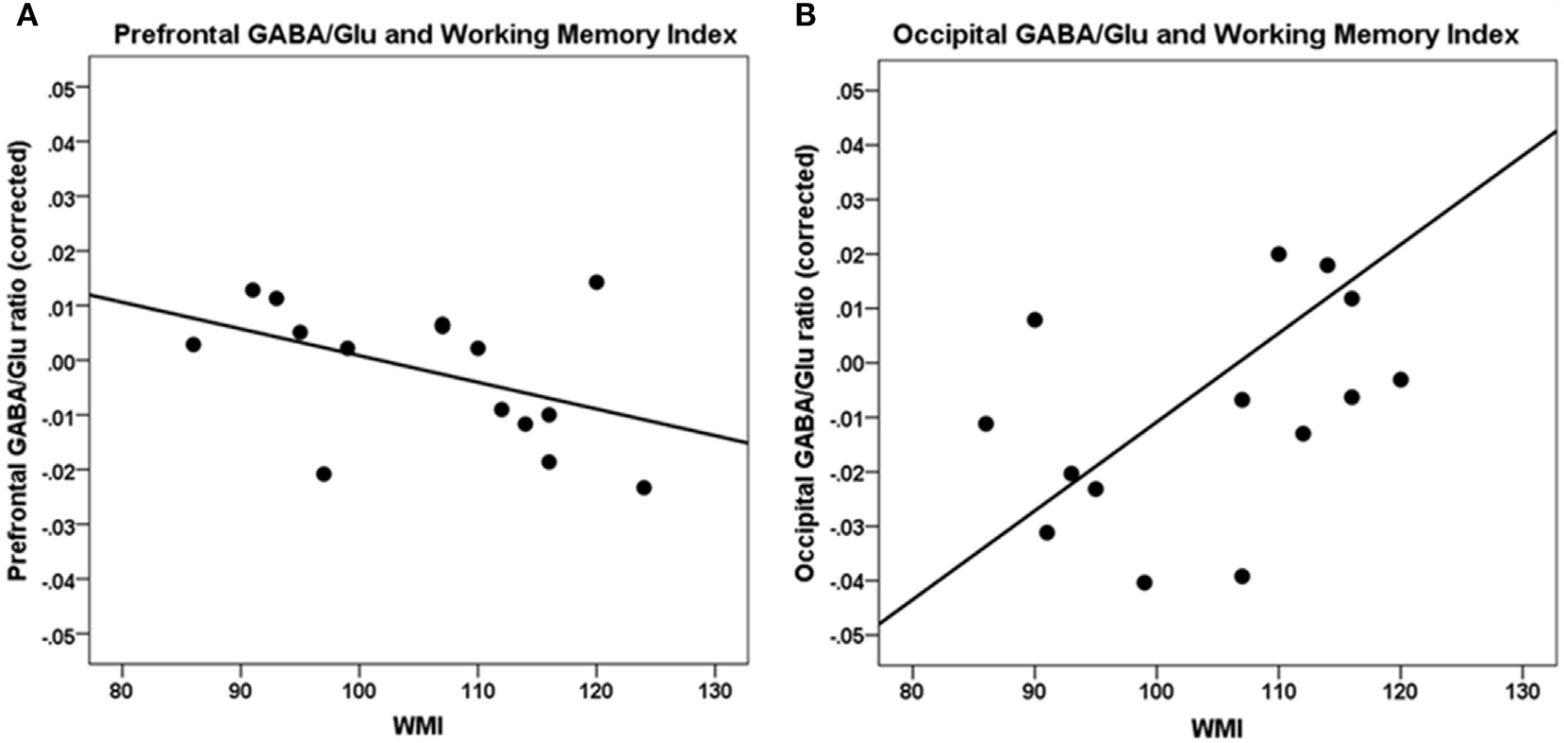
Correlations between working memory index (WMI) and GABA/Glu ratio. **(A)** Prefrontal GABA/Glu ratios are significantly correlated with WMI [*r*(7) = −0.80, *p* = 0.01]. **(B)** Occipital GABA/Glu ratios are not significantly correlated with WMI. Data are presented corrected for gray and white matter fractions, age, and sex.

A higher WMI was significantly correlated with a lower GABA/Glu ratio (GABA/Cr to Glu/Cr ratio) in the frontal cortex [*r*(7) = −0.80, *p* = 0.01] and not significantly in the occipital cortex [*r*(7) = 0.68, *p* = 0.04] (Table 2; Figure 3).

**Table 2:** Brain metabolites and intelligence.^a^

Metabolites	Cognition					
	TIQ	VIQ	PIQ	VCI	PRI	WMI
**Prefrontal**						
GABA/Cr	0.31	0.28	0.30	0.25	0.37	−0.05
Glutamate	−0.01	−0.25	0.26	0.01	0.28	−0.53
GABA/Glu	−0.51	−0.67	−0.19	−0.41	−0.13	−0.80**
**Occipital**						
GABA/Cr	0.03	0.09	−0.08	0.04	−0.18	0.42
Glutamate	−0.19	−0.27	−0.07	−0.17	−0.10	−0.79**
GABA/Glu	0.14	0.22	−0.06	0.05	−0.19	0.68*

*Bold font indicates significant correlations*. *^a^Pearson correlations, corrected for age, sex, and for local gray matter and white matter fractions*.

**p ≤ 0.01.

*p ≤ 0.05.

*TIQ, total intelligence quotient; VIQ, verbal intelligence quotient; PIQ, performance intelligence quotient; VCI, verbal comprehension index; PRI, perceptual reasoning index; WMI, working memory index*.

**Figure 4:**
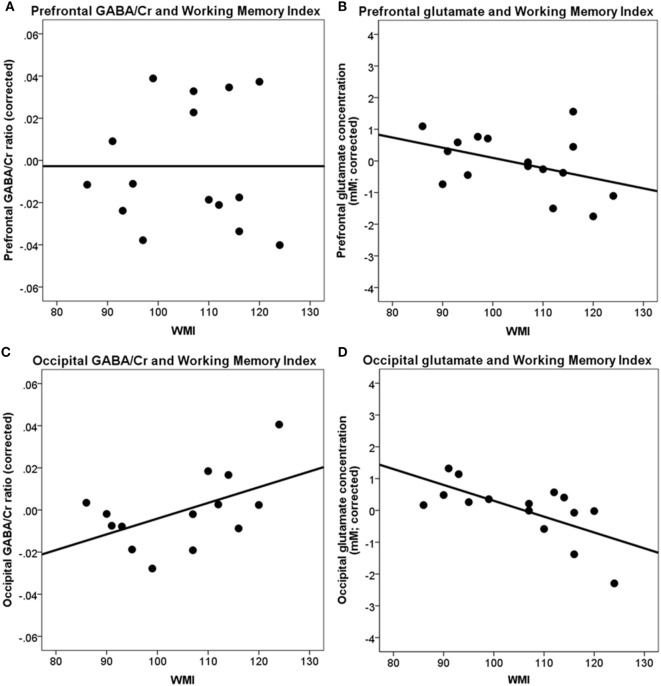
Correlations between prefrontal and occipital GABA/Cr and glutamate with working memory index (WMI). **(A)** Prefrontal GABA/Cr ratios are not significantly correlated with WMI; **(B)** prefrontal glutamate concentrations are not significantly correlated with WMI; **(C)** occipital GABA/Cr ratios are not significantly correlated with WMI; **(D)** occipital glutamate concentrations are significantly correlated with WMI [*r*(10) = −0.79, *p* < 0.004]. Data are presented corrected for gray and white matter fractions, age, and sex.

A higher WMI was not significantly correlated with the frontal GABA/Cr ratio [*r*(10) = −0.05, *p* = ns], frontal glutamate concentration [*r*(10) = −0.53, *p* = 0.076], and occipital GABA/Cr ratio [*r*(9) = 0.42, *p* = 0.19]. A higher WMI was significantly associated with a lower occipital glutamate concentration [*r*(10) = −0.79, *p* < 0.004] (Table 2; Figure 4).

No significant associations were found for TIQ, Verbal intelligence quotient, performance intelligence quotient, VCI, and PRI with any of the metabolites in the frontal and occipital cortices. All correlations with metabolites were corrected for age, sex, and gray and white matter fractions.

### Brain Metabolites, Age, and Sex

There were no other significant associations between metabolite levels in the prefrontal and occipital cortices with age. These correlations were corrected for sex, and gray and white matter fractions. There were no significant associations between metabolite levels in the prefrontal and occipital cortices with sex.

### Correlations among Metabolites

Following correction for age, sex, gray and white matter fractions, in the occipital cortex, a significant negative correlation was found between GABA/Cr ratio and glutamate concentration [*r*(7) = −0.85, *p* < 0.01].

**Figure 5:**
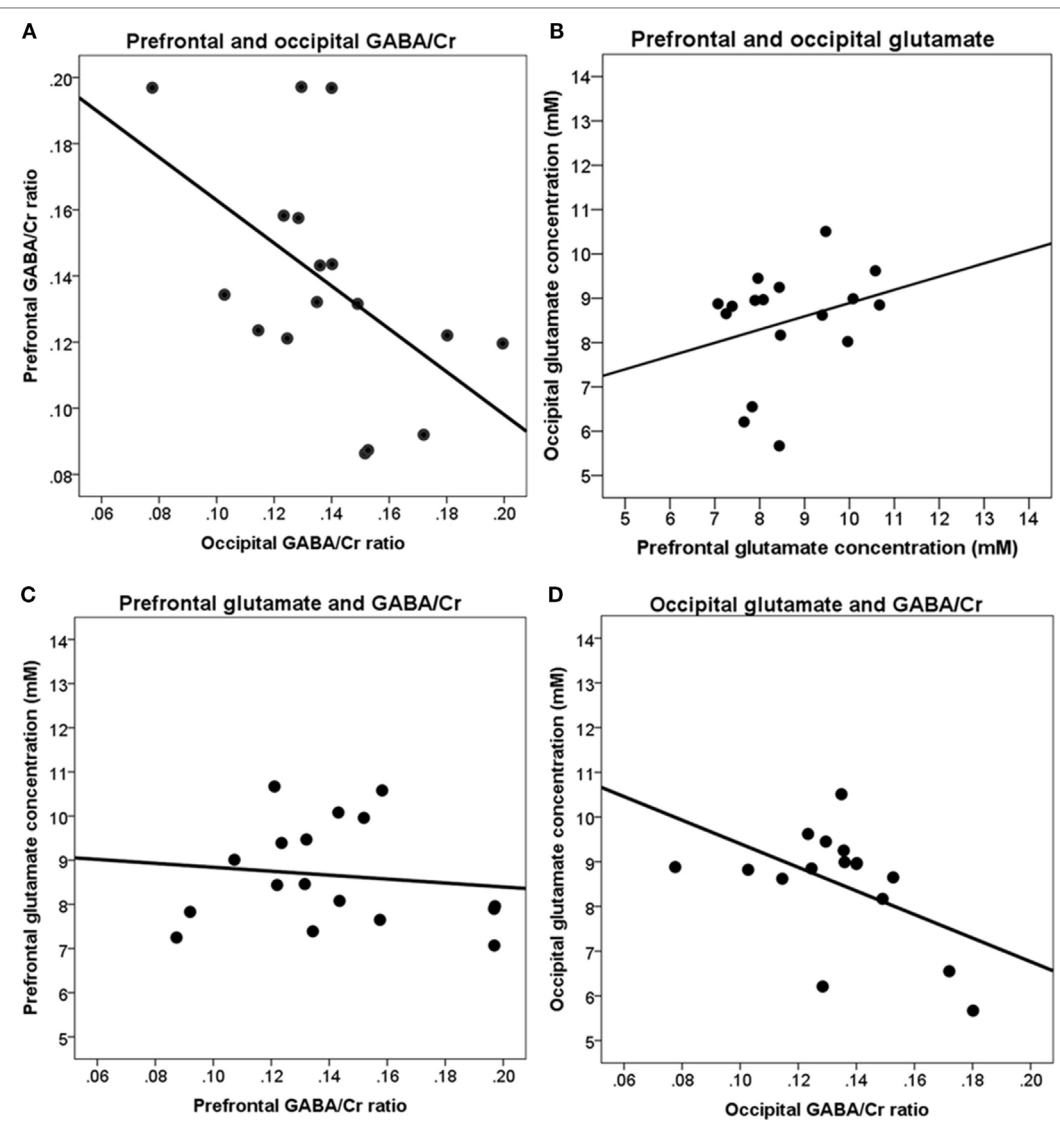
Correlations between brain metabolites in the prefrontal and occipital cortices. **(A)** Prefrontal and occipital GABA/Cr ratios are not significantly correlated; **(B)** prefrontal and occipital glutamate concentrations are not significantly correlated; **(C)** prefrontal GABA/Cr ratios and glutamate concentrations are not significantly correlated; **(D)** occipital GABA/Cr ratios and glutamate concentrations are significantly correlated [*r*(7) = −0.85, *p* < 0.01]. Data are presented uncorrected for gray and white matter fractions, age, and sex.

Higher GABA/Cr ratios in the prefrontal cortex were significantly correlated with lower glutamate concentrations [*r*(7) = −0.89, *p* < 0.01] in the occipital cortex (Figure 5).

## Discussion

To our knowledge, this study presents the first proton magnetic resonance spectroscopy measurements of GABA and glutamate levels *in vivo* at a magnetic field strength of 7 T associated with level of intelligence. The main finding is that a higher WMI is associated with a significantly lower GABA/Glu ratio in the frontal cortex. However, in contrast to what was expected, we also find a significantly lower glutamate concentration in the occipital cortex. This may suggest that the excitation–inhibition balance in the frontal cortex, and perhaps to a lesser extent in the occipital cortex, is associated with working memory. Working memory refers to the ability to actively hold information on-line over brief periods of time (41). Working memory is one of the factors marking intelligence and has been closely related to general intelligence, although the extent of overlap is point of discussion (42). Moreover, working memory has been positively associated with gray and white matter volume, and with white matter tracts, and these associations are under genetic control (3, 43). Interestingly, based on studies in animals, it has been found that a successful working memory performance requires an exquisite balance of the excitatory and inhibitory circuitry in the prefrontal cortex that includes glutamate and GABA (41). Neurons in the prefrontal cortex have been shown to fire persistently during the maintenance phase of working memory tasks for which a balance between inhibitory and excitatory neurons are thought to be required. Supportive evidence for such a system during activation in the human brain was found in a study where a lower resting-state GABA level was associated with higher amplitude of the BOLD fMRI response to a simple visual stimulus in the visual cortex (44) [for review, see Ref. (45)]. Other negative associations were found between resting-state GABA level with visual orientation discrimination performance in the occipital cortex (46), and in the supplementary motor area with tactile discrimination performance (47, 48), which also support associations between GABA levels in the human cortex with cognitive functioning. Here, we show, by using ^1^H-MRS at 7 T, that lower frontal resting-state GABA/Glu ratios and lower occipital glutamate concentrations may lead to a higher working memory performance in healthy adults, possibly through a more efficient inhibition–excitation balance.

Our measurements of brain metabolites were done in the frontal and occipital cortices using sLASER and MEGA-sLASER at 7 T. We could thus measure to which extend the metabolites were correlated within individuals. The most prominent association was found between levels of glutamate and GABA/Cr ratio in the occipital cortex, with higher levels of glutamate being associated with lower levels of GABA/Cr (−0.85). Interestingly, a significant negative correlation between GABA/Cr in the occipital cortex and glutamate level in the prefrontal cortex was also found, thus suggesting a possible differential and maybe connected resting state association between these two anatomically distant brain regions.

This study has some limitations to take into account. One, with MRS, one cannot distinguish intracellular and extracellular metabolite levels. Two, because of its low concentration, a large voxel size is needed to reliably and time-efficiently measure GABA. Hence, the voxel contained both gray and white matter. Three, the stringent correction for multiple comparisons was not possible due to the relatively limited number of participants that were included in the study. We chose for a more lenient approach to allow for the exploration of the association between these metabolite levels and intellectual functioning. Future studies with larger number of participants may allow for a more stringent correction for the multiple comparisons to confirm the stability of these findings.

Future studies using instance modern network analyses (49) may reveal how such connections between metabolite levels act. Such studies may reveal to which extent the associations between intelligence and brain metabolites are linked to local glucose levels and BOLD fMRI effects as well as to network efficiency of these nodes with the rest of the brain. There is evidence that the metabolic costs of a brain area (i.e., a node in a network) are proportional to the number of (mathematical) paths it has to connect with other nodes, and that the metabolic costs of a path are proportional to the physical distance it spans between nodes (50). Interestingly, Brodmann areas with a high glycolytic index were also found to be hub areas (i.e., have a high centrality rank); including Brodmann areas 32 and 33 (51), which are anatomically overlapping with the medial prefrontal voxel location of our current study. A functional neural network study revealed that a higher intelligence was associated with more efficient brain network (6), and thus possibly with a more efficient use of local brain metabolism including GABA and glutamate. Indeed, associations between GABA, glutamate, BOLD signal (52, 53), and functional connectivity (54) have been reported. A recent study found that the frontal GABA/Glx ratio (Glx is the sum of the glutamate and glutamine signals) is related to oscillatory modulations during a working memory task, showing that a low GABA/Glx ratio is needed for efficient inhibition of irrelevant neural activity in precise task performance (55). Changes in GABA and glutamate levels have been found in patients with schizophrenia, a disorder, which is known to affect cognitive functioning (22, 30, 56). Recent evidence supports the involvement of glutamate and GABA in working memory performance in schizophrenia (57).

In conclusion, we found that working memory performance is associated with the excitation–inhibition balance in the brain.

## Ethics Statement

This study was carried out in accordance with the recommendations of the institutional ethics board (METC) with written informed consent from all subjects. All subjects gave written informed consent in accordance with the Declaration of Helsinki. The protocol was approved by the institutional ethics board (METC).

## Author Contributions

AM was involved in the design of the study, data acquisition, data analysis, and writing of the manuscript. RM and HH were involved in the design of the study, data analysis, and writing of the manuscript. DK, WC, RK, and PL were involved in the design of the study and revising the manuscript.

## Conflict of Interest Statement

The authors declare that the research was conducted in the absence of any commercial or financial relationships that could be construed as a potential conflict of interest.
